# Predicting the Risk Factors Associated With Severe Outcomes Among COVID-19 Patients–Decision Tree Modeling Approach

**DOI:** 10.3389/fpubh.2022.838514

**Published:** 2022-05-19

**Authors:** Mahalakshmi Kumaran, Truong-Minh Pham, Kaiming Wang, Hussain Usman, Colleen M. Norris, Judy MacDonald, Gavin Y. Oudit, Vineet Saini, Khokan C. Sikdar

**Affiliations:** ^1^Surveillance and Reporting, Provincial Population and Public Health, Alberta Health Services, Calgary, AB, Canada; ^2^Surveillance and Reporting, Cancer Research and Analytics, Cancer Care Alberta, Alberta Health Services, Edmonton, AB, Canada; ^3^Division of Cardiology, Department of Medicine, University of Alberta, Edmonton, AB, Canada; ^4^Mazankowski Alberta Heart Institute, Faculty of Medicine and Dentistry, University of Alberta, Edmonton, AB, Canada; ^5^Faculty of Nursing, University of Alberta, Edmonton, AB, Canada; ^6^Cardiovascular Health and Stroke Strategic Clinical Network, Alberta Health Services, Edmonton, AB, Canada; ^7^Department of Community Health Sciences, Cumming School of Medicine, University of Calgary, Calgary, AB, Canada; ^8^Communicable Disease Control, Provincial Population and Public Health, Alberta Health Services, Calgary, AB, Canada; ^9^Department of Community Health Sciences and O'Brien Institute for Public Health, Cumming School of Medicine, University of Calgary, Calgary, AB, Canada; ^10^Public Health Evidence and Innovation, Provincial Population and Public Health, Alberta Health Services, Calgary, AB, Canada

**Keywords:** COVID-19, SARS-CoV-2, decision tree modeling, machine learning, outcome

## Abstract

**Background:**

The COVID-19 pandemic has seen a large surge in case numbers over several waves, and has critically strained the health care system, with a significant number of cases requiring hospitalization and ICU admission. This study used a decision tree modeling approach to identify the most important predictors of severe outcomes among COVID-19 patients.

**Methods:**

We identified a retrospective population-based cohort (*n* = 140,182) of adults who tested positive for COVID-19 between 5^th^ March 2020 and 31^st^ May 2021. Demographic information, symptoms and co-morbidities were extracted from a communicable disease and outbreak management information system and electronic medical records. Decision tree modeling involving conditional inference tree and random forest models were used to analyze and identify the key factors(s) associated with severe outcomes (hospitalization, ICU admission and death) following COVID-19 infection.

**Results:**

In the study cohort, nearly 6.37% were hospitalized, 1.39% were admitted to ICU and 1.57% died due to COVID-19. Older age (>71Y) and breathing difficulties were the top two factors associated with a poor prognosis, predicting about 50% of severe outcomes in both models. Neurological conditions, diabetes, cardiovascular disease, hypertension, and renal disease were the top five pre-existing conditions that altogether predicted 29% of outcomes. 79% of the cases with poor prognosis were predicted based on the combination of variables. Age stratified models revealed that among younger adults (18–40 Y), obesity was among the top risk factors associated with adverse outcomes.

**Conclusion:**

Decision tree modeling has identified key factors associated with a significant proportion of severe outcomes in COVID-19. Knowledge about these variables will aid in identifying high-risk groups and allocating health care resources.

## Introduction

The first outbreak of an acute respiratory disease caused by a novel coronavirus (SARS-CoV-2) was identified in Wuhan, China in December 2019. Following the continued spread of the coronavirus across several countries, the World Health Organization (WHO) declared a pandemic on 11^th^ March 2020 (1). As of 26^th^ March 2022, Canada has reported 3.45 million confirmed cases and 37,449 deaths (2). Globally the COVID-19 pandemic has resulted in approximately 479 million cases and 6.12 million deaths (1).

In Alberta, as of 26^th^ March 2022, approximately 536,166 confirmed cases and 4,044 deaths have been reported. Although most COVID-19 cases have presented with mild symptoms, there has been a relatively low rate of hospitalization (4.1%) and ICU admission (0.7%) noted in the province due to improving vaccination rates. However, there remains a critical need for understanding the epidemiological factors and clinical characteristics associated with severe outcomes in the planning and management of the disease.

Studies have reported that age, sex, smoking status, underlying health conditions, and clinical presentation including breathing difficulties, fever and cough are important risk factors associated with severe outcome(s) (3–8). Despite an increase in well-conducted prediction analysis with regression modeling, a direct translation of the research findings into patient care remains challenging. Decision tree modeling is a promising strategy for risk predictions based on a set of sequential rules. Decision tree models are intuitive, easy to interpret by non-statisticians and offer a new way to visualize complex datasets (9). This approach has demonstrated enhanced clinical utility in identifying high-risk individuals and aiding in subsequent intervention measures (10). Also, recent literature demonstrate that machine learning approaches have successfully predicted COVID-19 infection (11, 12) and outcomes based on clinical factors (disease severity), epidemiological factors age (13), sex, socioeconomic status, public health measures (lockdowns, restriction on gatherings, quarantine, social distancing measures), underlying health conditions, and presenting symptoms (14, 15).

Finally, the province of Alberta has experienced five waves of COVID-19 to date, with high numbers of new infections and deaths occurring in each wave. It is expected that there will be additional waves. It is, therefore, important to study the factors associated with severe outcomes to determine which factors are the most important to target for prevention and mitigation. Therefore, the aim of this study was to carry out a decision tree modeling approach to identify the risk factors associated with severe outcomes among COVID-19 patients in Alberta, Canada between March 2020 and May 2021 to support future clinical decision making and planning.

## Methods

### Ethics Approval

This study was approved by the Conjoint Health Research Ethics Board of Alberta (REB20-1257) at the University of Calgary which waived the requirement for informed consent.

### Study Cohort

In this retrospective population-based cohort study, we included all individuals in the province of Alberta who tested positive for SARS-CoV-2 infection from 5^th^ March 2020 to 31^st^ May 2021. All cases were laboratory confirmed for SARS-CoV-2 infection from clinical samples analyzed by real-time reverse transcriptase-polymerase chain reaction (rRT-PCR). In Alberta, the Communicable Disease and Outbreak Management (CDOM) information system is maintained by the provincial healthcare organization, Alberta Health Services (AHS). The CDOM system contains information obtained from the public health investigation of all cases of COVID-19 (confirmed and probable) in the province and reports these cases to the provincial health department as required by law. For this analysis, we extracted patient information pertaining to demographics and symptoms at the time of diagnosis (as reported in CDOM), and 3-year underlying conditions (as reported in administrative databases such as the Discharge Abstract Database (DAD), National Ambulatory Care Reporting System (NACRS) and Physician billing claims). Pre-existing conditions were grouped based on ICD codes (ICD 9/10 codes) (16) corresponding to the Charlson comorbidity index (16). The Pampalon index (17) for dissemination area levels from 2016 Census data was used to assign area-level material and social deprivation status based on the patient's postal code.

Charlson comorbidities were grouped for the analysis as follows: cardiovascular diseases (myocardial infarction, congestive heart failure, peripheral vascular disease); renal diseases; gastrointestinal (GI)/liver diseases (peptic ulcer disease, mild liver disease); pulmonary diseases (chronic pulmonary disease, asthma, chronic obstructive pulmonary disease); Neurological conditions (cerebrovascular disease, dementia, paraplegia); diabetes (diabetes with/without complications); cancer (immune-suppressive cancer, any malignancy and/or had previous cancer diagnosis); hypertension; and obesity (physician-diagnosed and self-reported conditions). Co-morbidities were considered as present using a cutoff of 2 years prior to the time of positive for COVID-19 laboratory confirmation.

The study cohort was restricted to adult patients only (>18 years of age) at the time of COVID diagnosis. The primary outcomes of the study were severity of disease as measured by hospitalization, ICU admission or death resulting from COVID-19 infection. Hospitalization or ICU admission within 30 days of onset of COVID-19 infection (symptom onset or laboratory test date if asymptomatic) or hospital-acquired COVID-19 infection were considered as an outcome. Cases that died from COVID-19 as a primary and/or secondary cause were considered as deaths.

For the analysis, disease severity was treated as an ordinal variable and assigned four possible values: 0 - cases who recovered without a need for hospitalization; 1 - hospitalized (but no ICU admission or death); 2 – ICU admission (but no death) and 3 - death (regardless of hospital/ICU admission).

To provide insights on age-specific risk factors, we have also performed age-stratified modeling in ages groups categorized as 18–40 years of age, 41–60 years of age and above 60 years of age.

### Decision Tree Modeling

Following data collection for the study cohort, the variables of interest – demographics, presenting COVID-19 symptoms at the time of diagnosis and underlying comorbidities were analyzed. We elected to use two different machine learning approaches to identify the consensus predictor variables.

#### Conditional Inference Trees (or CTREE)

The CTREE method was originally developed by Hothorn et al. (18), which is a decision tree modeling approach that recursively partitions the heterogenous study samples into homogenous subgroups. The partitioning is based on an exhaustive search within the input variables, followed by selecting the variables at each node which give the best split and performing a significance test at each node. At the terminal nodes, the outcome probabilities are calculated for the sample subgroup. We have used the CTREE algorithm that is built and implemented in the R package PARTY (18–20) to perform the present analyses. The study samples were randomly classified as training (~70%) and test (~30%) datasets. The outcome variable was an ordinal level variable and has 4 levels according to the disease severity (0 - COVID-19 case but no hospitalization/ICU/death; 1 - hospitalized (but no ICU or death); 2 - ICU admission (but no death) and 3 - death (regardless of hospital/ICU admission). Overall model accuracies and misclassification rate were calculated from the model.

Outcome (level = 0, 1, 2, 3) ~ age + Sex + Zone + Smoking_status + CVD + Renal + Gastrointestinal + Pulmonary + Neurological + Diabetes + Cancer + Other + Hypertension + Obesity + Fever + Breathing_difficulty + Chest_pain + Headache + Cough + Sore_throat + Pain + Gastrointestinal_symptoms + Nasal + Taste_smell + Other_symptoms.

#### Random Forest

Random forest is an unsupervised clustering algorithm that creates an ensemble of trees resulting in a forest. This is a machine learning approach implemented in the “ranger” package (21) optimized for a larger dataset. This method fits “*n*” classification trees by randomly selecting the independent variables for each tree to create an ensemble. In total, 500 different trees were created by bootstrapping and the best predictors were scored. To reduce the overfitting of the models, the datasets were classified into a training dataset (70% of the cases were used to build the model) and a test dataset (30% of the cases were used to make the prediction based on the training model). This model uses the out of bag (OOB) sampling approach to measure the prediction strength of each variable. For every tree that is grown, OOB samples are passed down and variables are randomly permuted. The model error rate is calculated based on the out-of-bag error and reported as the Brier score. Variable importance measurement is calculated as the Gini index. Following the estimation of the decision tree, they were pruned to reduce the number of sub nodes based on the importance.

#### Performance Metrics

Model performance metrics are indicated as accuracy or misclassification rate available for both the training and test dataset. In addition, we assessed the performance of the predictor variables using receiver operator curve (ROC) analysis. For the ROC analysis, we regrouped the multi-level outcome variable into a bivariate outcome (0- no hospitalization/ICU/death and 1- any event hospitalization/ICU/death) variable and performed the CTREE modeling with the same independent variables (Supplementary Section – Performance metrics). We used the “pROC” package in R. The R codes are available upon request to the authors.

## Results

Approximately 167,000 cases were identified in the study period prior to the spread of the Delta variant in Alberta. After excluding pediatric cases, a total of 140,182 adult cases who tested positive for COVID-19 were considered for further analysis. About 95% of cases recovered without any severe outcome. However, 6.37% were hospitalized, 1.39% were admitted to ICU and 1.57% died due to the disease. The frequency of variables used in the modeling is described in Table 1 including demographics, smoking status, underlying symptoms, and pre-existing conditions.

**Table 1:** Distribution of the variables by outcome in the study cohort.

**Category**	**Total cohort**	**Hospitalized**	**ICU**	**Death**
	**(*n* = 1,40,182)**	**(*n* = 8,943)**	**(*n* = 1,948)**	**(*n* = 2,199)**
**Outcomes**				
Hospitalized	8,943 (6.37%)			
Admitted to ICU	1,948 (1.39%)	1,948 (21.78%)		
Death	2,199 (1.57%)	1,328 (14.85%)	461 (23.67%)	
**Sex**				
Female	69,662 (49.69%)	3,973 (44.43%)	688 (35.32%)	973 (44.25%)
Male	70,520 (50.31%)	4,970 (55.57%)	1,260 (64.68%)	1,226 (55.75%)
**Age (years)**				
18 to <40 years	72,171 (51.48%)	1,560 (17.44%)	241 (12.37%)	16 (0.73%)
41 to <60 years	46,992 (33.52%)	2,765 (30.92%)	736 (37.78%)	160 (7.28%)
>60 years	21,019 (14.99%)	4,618 (51.64%)	971 (49.85%)	2,023 (92.0%)
**Ethnicity**				
Caucasian	65,142 (46.47%)	3,810 (42.6%)	764 (39.22%)	730 (33.2%)
Asian	22,956 (16.38%)	1,069 (11.95%)	265 (13.6%)	194 (8.82%)
African	8,924 (6.37%)	355 (3.97%)	73 (3.75%)	21 (0.95%)
Other**	43,160 (30.79%)	3,709 (41.47%)	846 (43.43%)	1,254 (57.03%)
**Geography**				
Edmonton zone	45,485 (32.45%)	3,400 (38.02%)	668 (34.29%)	1,084 (49.3%)
Calgary zone	57,769 (41.21%)	2,945 (32.93%)	702 (36.04%)	672 (30.56%)
All other*	36,928 (26.34%)	2,598 (29.05%)	578 (29.67%)	443 (20.15%)
**Smoking status**				
Never smoked	1,16,242 (82.92%)	6,862 (76.73%)	1,434 (73.61%)	1,799 (81.81%)
Past smokers	9,395 (6.7%)	1,266 (14.16%)	324 (16.63%)	326 (14.82%)
Current smokers	14,545 (10.38%)	815 (9.11%)	190 (9.75%)	74 (3.37%)
Underlying condition				
Cardiovascular disease	6,987 (4.98%)	2,136 (23.88%)	413 (21.2%)	1,042 (47.39%)
Renal disease	3,630 (2.59%)	1,354 (15.14%)	296 (15.2%)	580 (26.38%)
Gastrointestinal/liver disease	4,944 (3.53%)	1,102 (12.32%)	277 (14.22%)	336 (15.28%)
Pulmonary disease	17,364 (12.39%)	2,503 (27.99%)	530 (27.21%)	751 (34.15%)
Hypertension	28,167 (20.09%)	4,914 (54.95%)	1,127 (57.85%)	1,634 (74.31%)
Neurological conditions	7,095 (5.06%)	1,720 (19.23%)	212 (10.88%)	1,216 (55.3%)
Diabetes	11,898 (8.49%)	2,827 (31.61%)	730 (37.47%)	780 (35.47%)
Cancer	2,428 (1.73%)	660 (7.38%)	138 (7.08%)	241 (10.96%)
Obesity	8,756 (6.25%)	1,434 (16.03%)	481 (24.69%)	243 (11.05%)
other	2,080 (1.48%)	325 (3.63%)	70 (3.59%)	89 (4.05%)
**Symptoms**				
Fever	54,581 (38.94%)	3,960 (44.28%)	1,069 (54.88%)	690 (31.38%)
Dyspnea	13,649 (9.74%)	3,867 (43.24%)	1,131 (58.06%)	803 (36.52%)
Chest pain	5,865 (4.18%)	735 (8.22%)	199 (10.22%)	63 (2.86%)
Headache	54,825 (39.11%)	2,444 (27.33%)	544 (27.93%)	171 (7.78%)
Cough	65,315 (46.59%)	5,110 (57.14%)	1,241 (63.71%)	937 (42.61%)
Sore throat	40,869 (29.15%)	1,713 (19.15%)	398 (20.43%)	177 (8.05%)
Myalgia/arthralgia	59,612 (42.52%)	3,986 (44.57%)	962 (49.38%)	579 (26.33%)
Gastrointestinal symptoms	26,761 (19.09%)	2,968 (33.19%)	712 (36.55%)	458 (20.83%)
Nasal symptoms	54,787 (39.08%)	1,968 (22.01%)	404 (20.74%)	234 (10.64%)
Loss of taste/smell	25,074 (17.89%)	913 (10.21%)	204 (10.47%)	54 (2.46%)
Other symptoms	23,623 (16.85%)	2,655 (29.69%)	657 (33.73%)	687 (31.24%)
**Material deprivation index**				
1 (Least deprived)	23,456 (16.73%)	1,149 (12.85%)	235 (12.06%)	262 (11.91%)
2	23,362 (16.67%)	1,188 (13.28%)	256 (13.14%)	172 (7.82%)
3	24,549 (17.51%)	1,338 (14.96%)	320 (16.43%)	234 (10.64%)
4	25,744 (18.36%)	1,630 (18.23%)	381 (19.56%)	299 (13.6%)
5 (Most deprived)	32,976 (23.52%)	2,381 (26.62%)	574 (29.47%)	400 (18.19%)
Missing***	10,095 (7.2%)	1,257 (14.06%)	182 (9.34%)	832 (37.84%)
**Social deprivation index**				
1 (Least deprived)	23,464 (16.74%)	1,304 (14.58%)	337 (17.3%)	173 (7.87%)
2	24,200 (17.26%)	1,207 (13.5%)	253 (12.99%)	222 (10.1%)
3	25,455 (18.16%)	1,380 (15.43%)	320 (16.43%)	213 (9.69%)
4	26,767 (19.09%)	1,650 (18.45%)	358 (18.38%)	342 (15.55%)
5 (Most deprived)	30,201 (21.54%)	2,145 (23.99%)	498 (25.56%)	417 (18.96%)
Missing***	10,095 (7.2%)	1,257 (14.06%)	182 (9.34%)	832 (37.84%)

* *Included North, Central and South zone of Alberta Health Services*.

** *Indicates the cases with minority or missing data for ethnicity*.

*** *These dissemination areas had missing material and social deprivation scores in the 2016 Pampalon Deprivation Index database*.

All variables (except deprivation index and ethnicity) were tested using for the ordinal outcome of disease severity (admission to hospital or ICU, or death) in the decision tree models. There were data quality issues with ethnicity information and missing deprivation index values for certain postal codes. Postal codes for long term care facilities/senior care facilities, which had higher case numbers and mortality rates, were excluded from the deprivation index value calculations. Therefore, we could not assign a deprivation index to cases that may have been residents of elder care facilities. These two variables were excluded in decision tree modeling to minimize bias in the models.

### CTREE Model Predicts the Risk Factors for Severe COVID-19 Related Outcomes for All Age Groups

**Figure 1:**
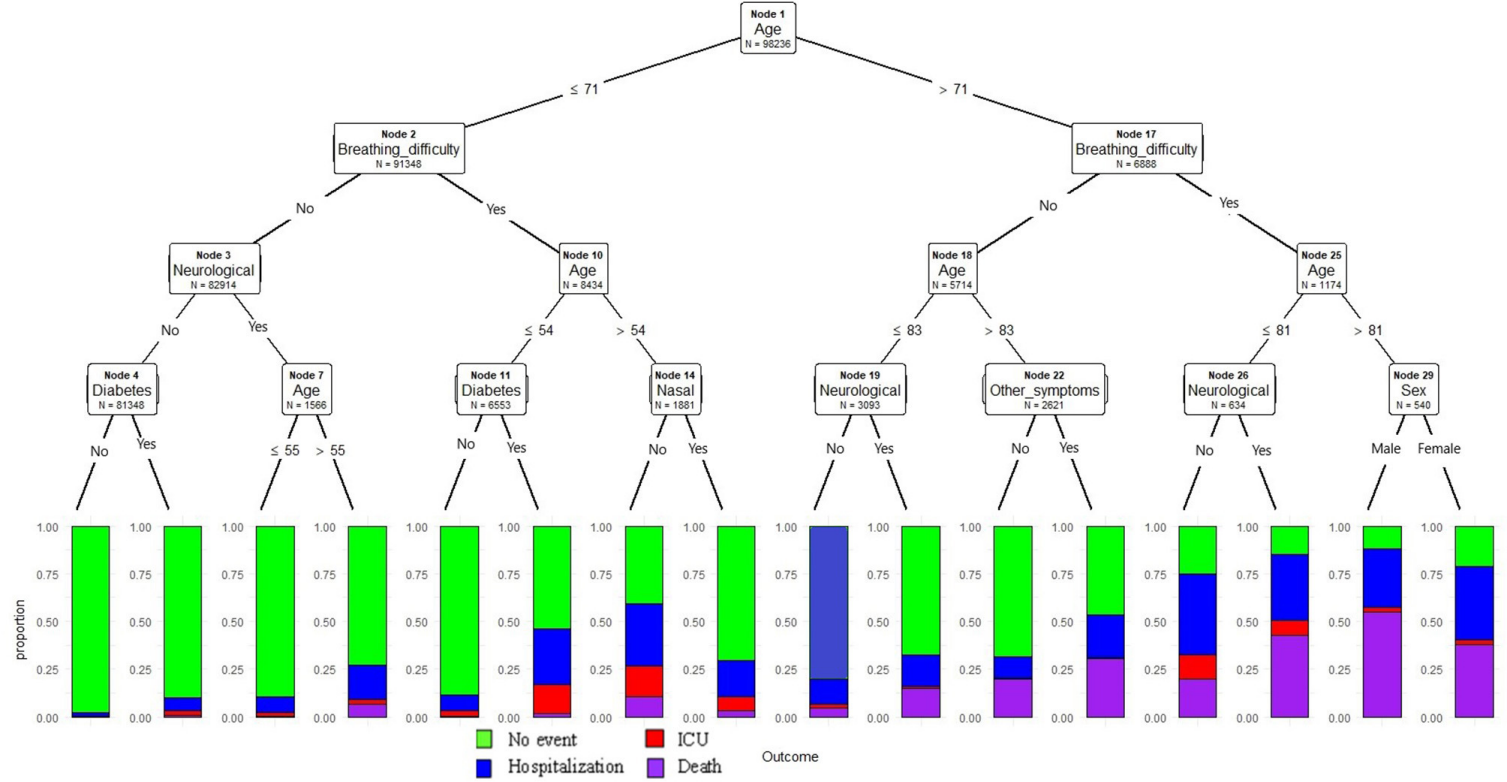
Conditional inference decision tree for classifying severe outcomes (hospitalization, ICU admission death) among COVID-19 positive cases. The outcomes are described in color codes (green color- no hospitalization, ICU or death; blue- hospitalization only, no ICU or death; red- admitted to ICU, but no death; purple -death). The proportion of the events were plotted on the Y-axis at each node.

In this model, we have classified all study samples into training (70%) and test datasets (30%). Age, breathing difficulty and neurological conditions were found to be the top three variables playing an important role in predicting outcomes, with a *p*-value <0.001 at each node. In this model, age was the continuous variable and resulting model estimates as described in Figure 1, age is selected at the primary node, with age >71 years followed by breathing difficulties node 17 increased the probability of death at nodes 27, 28, 30, and 31 compared to other nodes in the model. At node 29, sex was identified as an important predictor known to be associated with severe outcomes in COVID; Males demonstrated a higher probability of death compared to females. Similarly at node 26, indicating the presence of neurological conditions was clearly associated with a higher probability of death. In contrast, without any breathing difficulties or neurological conditions or diabetes (nodes 5) demonstrated lower probability of the severe outcomes compared to node 6 where presence of diabetes increased the probability of hospitalization. Similarly, cases with breathing difficulty and among cases ≤ 54 years (node 13), the presence of diabetes increased the hospitalization probability.

The accuracy of the model based on the training dataset (*n* = 98,236) was 93.3% and outcome prediction based on the test data (*n* = 41,936) had an accuracy rate of 93.2%.

**Figure 2:**
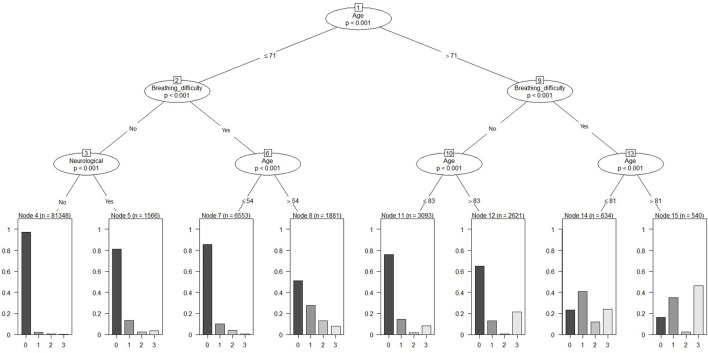
Pruned conditional inference decision tree for classifying severe outcomes (hospitalization, ICU admission or death) among COVID-19.

The above model was pruned to a maximum depth = 3, and thereby resulting in a model with a smaller number of features but similar prediction accuracy compared to the unpruned models. Age, breathing difficulties and neurological conditions were the top predictors of the outcome (Figure 2). Age > 71 years and presence of breathing difficulties had a probability of poor outcome compared to the group without breathing difficulties. Overall, older age and the presence of breathing difficulties demonstrated a trend of increased probability of hospitalization, ICU admission or death.

### CTREE Modeling by Age Category

In our primary CTREE model (discussed above), we have used age as a continuous variable and allowed the CTREE algorithm to select an appropriate cut-off for the split at each node. Based on our findings and evidence from the literature, age is an important predictor of outcome. However, the prevalence of comorbidities varies widely across the age group. Therefore, to understand the significance of the specific set of comorbidities and their role in the outcome in different age groups, we performed an additional stratified analysis based on age group categories 18–40 Y, 41–60 Y, and > 60 Y. The age-stratified modeling approach provided insight into the risk factors within the particular age groups.

#### Age Group 18–40 Y

We performed the analysis on the subgroup of cases with age <40 years (*n* = 72,171) (Table 2 and Supplementary Figure 1A). The dataset was split into training (*n* = 50,610) and test data sets (*n* = 21,561) and we included age as a continuous variable, this will allow the model to select the high-risk age cut-off within the age-stratified group. The model identified that age, breathing difficulty, diabetes, and obesity as the top predictive factors. Cases with breathing difficulties had higher hospitalization rates and ICU admission compared to cases without breathing difficulties. Among the cases without breathing difficulties, the presence of cardiovascular disease or CVD (node 5& 8) and renal disease (node 9) resulted in a higher probability of severe outcomes. The prediction accuracy for this classification model is 98%.

**Table 2:** CTREE and random forest model comparison.

		**CTREE**		**Random forest**		
**Ordinal outcome (0, 1, 2, 3)**	**Sample size**	**Top 4 variables**	**Accuracy**	**Top 4 variables**	**Percentage of the total outcome predicted based on top 4 variables**	**Accuracy (Brier Score)**
All ages	1,40,182	• Age • breathing difficulty • neurological conditions • Diabetes	93.3%	• Age (28%), • breathing difficulty (22%), cardiovascular disease (8%), • neurological conditions (7%)	65 %	94%
18–40 Y	72,171	• Breathing difficulty • Obesity • Diabetes • Cardiovascular disease	98%	• Breathing difficulty (15.7%) • Diabetes (10.12%) • Age (9.65%) • Obesity (6.76%)	42%	98%
41–60 Y	46,992	• Breathing difficulty • Diabetes • Renal • Nasal	94%	• Breathing difficulty (33.2%) • Age (7%) • Diabetes (6.4%) • Nasal (4.96%)	51.63%	96%
>60 Y	21,019	• Breathing difficulty • Age • Neurological condition • Nasal	76%	• Breathing difficulty (31.4%) • Age (17.4%) • Neurological condition (5.7%) • Cardiovascular disease (5.3%)	60%	80%

#### Age Group 41–60 Y

In this 41–60 Y age group stratum of 46992 cases, the dataset is split into a training (*n* = 32,956) and test (*n* = 14,036) (Table 2 and Supplementary Figure 1B). The prediction accuracy for this classification model was 94%. Breathing difficulties, diabetes and renal disease were the top three predictors for this age group. Cases with breathing difficulty and diabetes along with other symptoms (node 27) or age >56 (node 24), and obesity (node 17) had higher probabilities of severe outcomes. Among cases without breathing difficulties, renal conditions, and CVD (node 10) increased the probabilities of all three severe outcomes.

#### Age Group Above 60 Y

There were 21,019 cases above the age of 60 years; this age group had more severe outcomes compared to the two other age groups. The training dataset included 14,731 cases and 6,288 cases were in the test dataset (Table 2 and Supplementary Figure 1C). The prediction accuracy for this model is 76%. Breathing difficulty and age were the top two predictors. At node 25, cases with breathing difficulty and age >84 had the highest mortality rate. Cases with breathing difficulties had increased probabilities of severe outcomes (right side of the tree) compared to cases without breathing difficulty.

### Top Predictors by CTREE Method Validated by Independent Random Forest Approach

In addition to the CTREE modeling, we utilized a random forest approach to validate the predictions. We performed the random forest modeling for datasets including all ages as well as samples stratified by age groups.

#### All Ages

**Figure 3:**
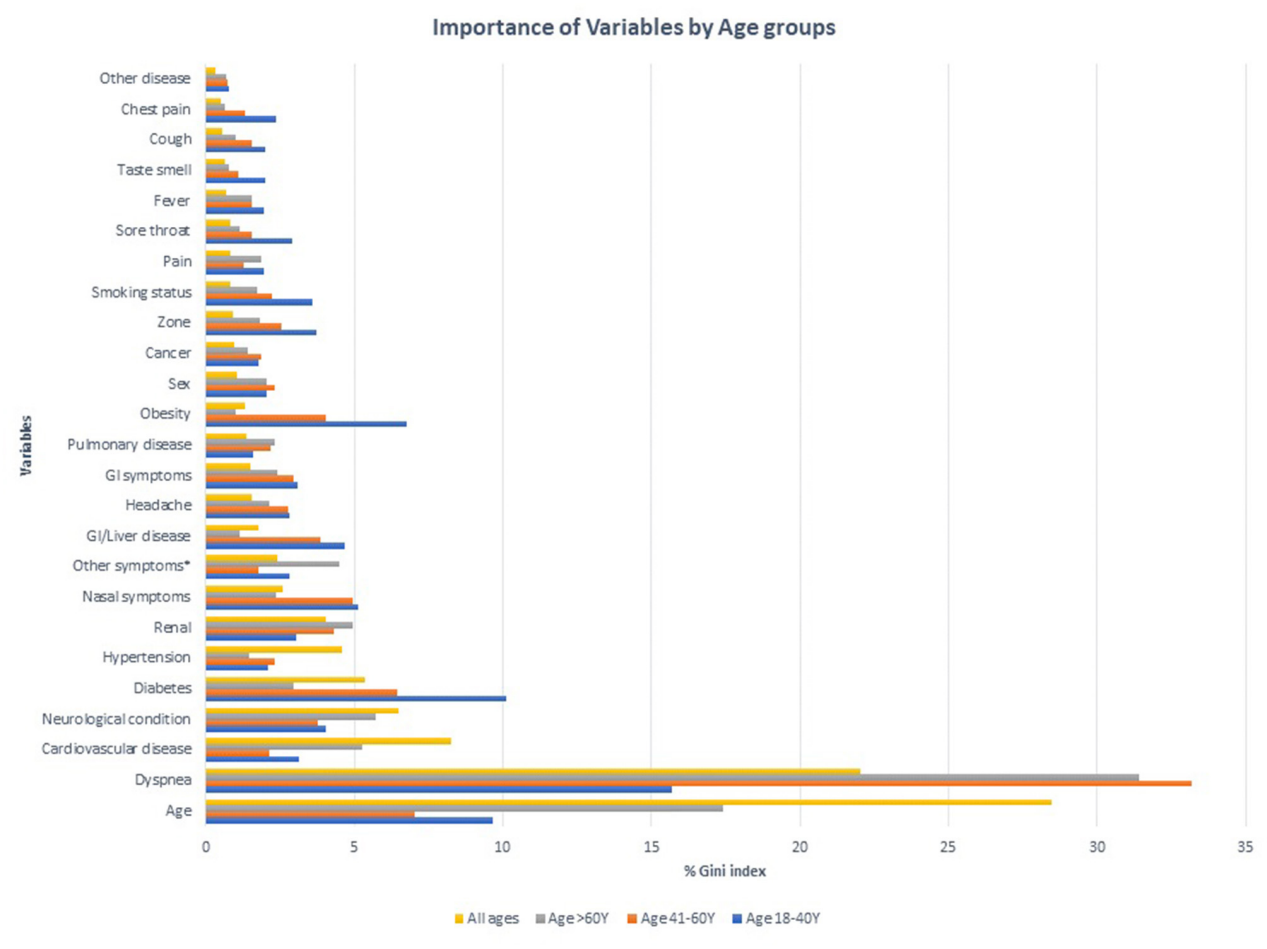
Importance of Variables in predicting severe outcomes among COVID-19 positive cases in different age groups.

According to the random forest model prediction based on the dataset that includes all ages and age variable is continuous presented in Figure 3. Based on this model, nearly 79% of the cases with severe outcomes can be predicted based on the top variables, about 50% of severe outcomes can be predicted based on age (28%) and symptoms of breathing difficulties (22%), and the five underlying conditions: cardiovascular conditions (8%), neurological conditions (7%), diabetes (5%), hypertension (5%), renal disease (4%) contribute to the model. The remaining symptoms and demographic predictors each contributed from 3% to <1%. The out of bag prediction error for the random forest model was about 6.68%. A comparison of the prediction errors from the conditional inference tree and decision tree modeling demonstrated ~93% accuracy in both techniques. The top two variables selected in CTREE, and random forest approaches were the same and the order of other variables was negligible.

#### Stratified Analysis by Age Group

Using the random forest approach in the age-stratified modeling of datasets identified different combinations of predictors than those described in Figure 3 and Table 2. In the model including cases with 18–40 Y age, breathing difficulties was the top variable predicting 16% of severe outcomes, with diabetes (10%), age (10%), obesity (7%) and represented a total of 42% of the outcomes among this age group. The prediction accuracy using this dataset was 98%.

Similarly in the group 41–60 Y, breathing difficulties (33%), age (7%), diabetes (6%), and nasal symptoms (%) were the top four predictors, collectively predicting 52% of the outcomes with a 96% accuracy.

The modeling used in the cohort >60 Y has consistently identified breathing difficulties (31%), age (17%), neurological conditions (6%), CVD (5%) as the top variables predicting nearly 60% of the outcomes in the group with an accuracy of 80%.

Irrespective of the age categories, breathing difficulty and age are the top two predictors consistent with other models.

### Model Performance

The performance of the model was by the model accuracy or misclassification rate. For both CTREE and random forest models, the accuracy of the models is indicated in Table 2. The generally predictive or discriminatory power of the independent variables is given by the area under the curve estimated from ROC analysis. As a ROC analysis requires bivariate outcomes; therefore, we have regrouped the multiclass outcome variable into bivariate outcomes (0-no event, 1-any hospital/ICU/Death). CTREE models were performed with the same independent variables as discussed above with a bivariate outcome. The CTREE models are illustrated in Supplementary Figures 2A–D. ROC plots are presented in Supplementary Figure 3 and AUC estimates for the models are available in Supplementary Table 2. The AUC for the model including all age groups has the highest discrimination at 0.83 [95% CI 0.82**–**0.84] compared to the age-stratified models.

## Discussion

In this population-based retrospective study of 1,40,182 cases of COVID-19, about 7% experienced severe outcomes such as hospitalization, ICU admission or death. We have adopted the Charlson comorbidity index approach to group the underlying conditions and use in the model to evaluate the association with COVID-19 disease severity. Similar approach has been adopted in other studies to identify the comorbidities associated with COVID-19 in different populations (22–24).

There is emerging literature evidence related to implementing machine learning approaches to understand the pattern and key contributors to COVID-19 infections and prognosis. This current study's findings that included one of the largest population-based cohorts and explored the predictors of specific outcomes such as hospitalization, ICU admission or death are consistent with literature (8, 25).

In the CTREE model including cases in all age groups, the key factors influencing severe outcomes were age, breathing difficulties, and the presence of pre-existing conditions, including cardiovascular disease, neurological conditions, diabetes, hypertension, and renal disease. Similarly, the random forest model identified that age and breathing difficulties were the two primary risk factors in predicting poor prognosis accounting for up to 28% and 22% of outcomes, respectively. Cardiovascular disease (8%) and neurological conditions (7%) were also noted as key predictors of outcome. About 65% of the outcomes could be predicted based on these four factors. The presence of diabetes (5%), hypertension (5%) and renal disease (4%) together with the top four factors predicted about 79% of the outcomes. Therefore, these variables are key to developing a decision rule. The results from the model can help clinicians quickly identify and prioritize high risk patients and allocate health care resources more effectively.

We also adopted age-stratified modeling to get a deeper insight into the risk factors within specific age groups. We consistently noticed that irrespective of age-based stratification, that breathing difficulties and age were critical in predicting severe outcomes. Interestingly, among the younger age group (18–40 Y) obesity was among the top predictors, consistent with literature findings (26) and adding critical knowledge. Diabetes, cardiovascular and neurological conditions were identified as predictive across the age stratified models.

Our findings are consistent with the current literature, where the majority of poor outcomes are noted among the older populations (3, 8, 25) and individuals with underlying conditions (6). A model based on age and other clinical variables predicted death among COVID-19 patients in China (8). Population prevalence of neurological conditions, which includes cerebrovascular disease, dementia, paraplegia, the chronic neurological disorder is higher among older adults (27). This is consistent with our findings, older age and neurological conditions together had the highest proportion of severe outcomes. Cardiovascular conditions and diabetes, which were among the top list of predictive variables in our models, have also been reported to play an important role in predicting patient outcomes in several independent studies (28).

In this study, older age with the presence of breathing difficulties demonstrated a higher rate of hospitalization when compared to older age without breathing difficulties. Similarly, older age with any of the above-mentioned preexisting conditions showed a clear pattern of increased hospital admission. Cases that are younger or without any preexisting conditions or breathing difficulties had the best prognosis. Also, consistent with the literature, cases in our cohort who had breathing difficulties irrespective of age or presence of preexisting conditions had higher rates of hospitalization (7, 29). Modeling studies based on different cohorts have shown age is a key factor associated with poor outcomes. Our findings are consistent with the literature evidence (8, 25) in that older male have a higher risk of dying compared to their female counterparts.

This study's identification of key factors in predicting COVID-19 disease severity is limited by the available data on variables of interest. In spite of evidence in the literature suggesting the importance of ethnicity and material/social deprivation index of cases in the context of COVID outcomes, we were unable to include these variables due to data quality issues related to missing data. Ethnicity information was inconsistently collected from cases, therefore excluded. In general, deprivation index calculation excludes postal codes of long-term care facilities or other seniors' facilities due to higher mortality rates compared to the rest of the geographies. Due to the unavailability of the deprivation indices for those postal codes, (older adults), the use of the deprivation index in the analysis may have biased the models. Therefore, we excluded the deprivation index in the model prediction analysis.

We also note that subsequent to the period of analysis in our study, the Omicron wave started in late November 2021 in Alberta, Canada. The transmissibility of the Omicron variants was known to be high compared to the variants of previous waves. There are studies suggesting the clinical profile of the Omicron infected patients showed decreased severity of disease compared to the previous waves. However, we were not able to confirm this using our public health data. As such, future modeling approaches should consider the variant type as a potential predictor of outcomes.

Vaccination has reduced severe outcomes of COVID-19 in the population and is also an important factor to consider in predictive models. Vaccine rollout in Alberta began in December 2020 based on strict eligibility criteria. Nearly 50% of cases in our study population had acquired COVID-19 infection in the initial stages of the pandemic before the vaccine rollout in Alberta, so vaccination status was not used as a predictor in our analysis. Interestingly, extraction of vaccination status among the cases in the period of the study showed that only a small proportion of cases had COVID-19 infection after immunization and also had an event of hospitalization, ICU admission or death. Among the cases with severe outcomes, approximately 3% had acquired infection following the first dose and 0.3% cases following the second dose.

Overall, the study model including all age groups as well age-stratified models clearly demonstrate that age and breathing difficulties are key predictive factors leading to severe outcomes among the COVID-19 cases. Underlying conditions including neurological conditions, diabetes, cardiovascular disease, hypertension, and renal disease increase the risk for the severe outcome. Interestingly among the younger adult's cohort, obesity was identified as an important predictor of severe outcomes. Ultimately, we found decision tree modeling approaches have great promise in identifying and informing the clinical management of high-risk COVID-19 patients.

## Data Availability Statement

The datasets presented in this article are not readily available because the data set from this study is held securely in coded and de-identified form at Alberta Health Services (AHS). Although data sharing agreements prohibit AHS from making the data set publicly available, access may be granted to those who meet pre specified criteria for confidential access. Please contact research.administration@ahs.ca for more information. Requests to access the datasets should be directed to research.administration@ahs.ca.

## Ethics Statement

The studies involving human participants were reviewed and approved by Conjoint Health Research Ethics Board of Alberta at the University of Calgary. Written informed consent for participation was not required for this study in accordance with the national legislation and the institutional requirements.

## Author Contributions

KS conceptualized, designed the study, acquired the data, and provided inputs on the analysis. MK conducted the analysis, interpreted the findings, and drafted the manuscript. KW and T-MP provided critical feedback on the analysis and drafting of the manuscript. VS, JM, GO, CN, and HU revised the manuscript critically for important intellectual content. All authors of this study contributed to this manuscript, have approved the manuscript for submission and have granted the corresponding author, and permission to submit the article for publication.

## Conflict of Interest

The authors declare that the research was conducted in the absence of any commercial or financial relationships that could be construed as a potential conflict of interest.

## Publisher's Note

All claims expressed in this article are solely those of the authors and do not necessarily represent those of their affiliated organizations, or those of the publisher, the editors and the reviewers. Any product that may be evaluated in this article, or claim that may be made by its manufacturer, is not guaranteed or endorsed by the publisher.

## References

[1] WHO. World Health Organization (WHO) *COVID*−*19 Situation Reports*. Geneva: World Health Organization (2020).

[2] Canada PHAO. COVID-19 Daily Epidemiology Update. Ottawa, ON: Public Health Agency of Canada (2020).

[3] Kang SJ Jung SI. Age–related morbidity and mortality among patients with COVID−19. Infect Chemother. (2020) 52:154–64. 10.3947/ic.2020.52.2.154 32537961 PMC7335648

[4] Gold JAW Wong KK Szablewski CM Patel PR Rossow J da Silva J. Characteristics and clinical outcomes of adult patients hospitalized with COVID−19 – georgia. MMWR Morb Mortal Wkly Rep. (2020) 69:545–50. 10.15585/mmwr.mm6918e1 32379729 PMC7737948

[5] Gebhard C Regitz–Zagrosek V Neuhauser HK Morgan R Klein SL. Impact of sex and gender on COVID−19 outcomes in Europe. Biol Sex Differ. (2020) 11:29. 10.1186/s13293-020-00304-9 32450906 PMC7247289

[6] Choi YJ Park JY Lee HS Suh J Song JY Byun MK. Variable effects of underlying diseases on the prognosis of patients with COVID−19. PLoS ONE. (2021) 16: 254258. 10.1371/journal.pone.0254258 34280188 PMC8289057

[7] He XB Cheng X Feng XD Wan H Chen SH Xiong MM. Clinical symptom differences between mild and severe covid−19 patients in china: a meta–analysis. Front Public Health. (2021) 8:561264. 10.3389/fpubh.2020.561264 33520906 PMC7841395

[8] Yang Q Li JX Zhang ZJ Wu XC Liao TQ Yu SY. Clinical characteristics and a decision tree model to predict death outcome in severe COVID−19 patients. Bmc Infect Dis. (2021) 21:783. 10.1186/s12879-021-06478-w 34372767 PMC8351764

[9] Piper ME Loh WY Smith SS Japuntich SJ Baker TB. Using decision tree analysis to identify risk factors for relapse to smoking. Subst Use Misuse. (2011) 46:492–510. 10.3109/10826081003682222 20397871 PMC2908723

[10] Perlich C Provost F Simonoff JS. Tree induction vs. logistic regression: a learning–curve analysis. J Mach Learn Res. (2004) 4:211–55.

[11] Wang PP Zheng XQ Li JY Zhu BR. Prediction of epidemic trends in COVID−19 with logistic model and machine learning technics. Chaos Soliton Fract. (2020) 139. 10.1016/j.chaos.2020.110058 32834611 PMC7328553

[12] Yang ZF Zeng ZQ Wang K Wong SS Liang WH Zanin M. Modified SEIR and AI prediction of the epidemics trend of COVID−19 in China under public health interventions. J Thorac Dis. (2020) 12:165. 10.21037/jtd.2020.02.64 32274081 PMC7139011

[13] Myers LC Parodi SM Escobar GJ Liu VX. Characteristics of hospitalized adults with COVID−19 in an integrated health care system in California. JAMA. (2020)323:2195–8. 10.1001/jama.2020.7202 32329797 PMC7182961

[14] Zoabi Y Deri–Rozov S Shomron N. Machine learning–based prediction of COVID−19 diagnosis based on symptoms. NPJ Digit Med. (2021) 4:3. 10.1038/s41746-020-00372-6 33398013 PMC7782717

[15] Wynants L Van Calster B Collins GS Riley RD Heinze G Schuit E. Prediction models for diagnosis prognosis of covid−19: systematic review critical appraisal. BMJ. (2020) 369:m1328. 10.1136/bmj.m1328 32265220 PMC7222643

[16] Quan H Sundararajan V Halfon P Fong A Burnand B Luthi JC. Coding algorithms for defining comorbidities in ICD−9–CM and ICD−10 administrative data. Med Care. (2005) 43:1130–9. 10.1097/01.mlr.0000182534.19832.83 16224307

[17] Pampalon R Hamel D Gamache P Raymond G. A deprivation index for health planning in Canada. Chronic Dis Can. (2009) 29:178–91. 10.24095/hpcdp.29.4.05 19804682

[18] Hothorn T Hornik K Zeileis A. Unbiased recursive partitioning: A conditional inference framework. J Comput Graph Stat. (2006) 15:651–74. 10.1198/106186006X13393333619988

[19] Strobl C Boulesteix AL Zeileis A Hothorn T. Bias in random forest variable importance measures: Illustrations, sources and a solution. Bmc Bioinformatics. (2007) 8:25. 10.1186/1471-2105-8-25 17254353 PMC1796903

[20] Zelleis A Hothorn T Hornik K. Model–based recursive partitioning. J Comput Graph Stat. (2008) 17:492–514. 10.1198/106186008X319331

[21] Breiman L. Random forests. Mach Learn. (2001) 45:5–32. 10.1023/A:1010933404324

[22] Christensen DM Strange JE Gislason G Torp–Pedersen C Gerds T Fosbol E. Charlson comorbidity index score and risk of severe outcome and death in danish COVID−19 patients. J Gen Intern Med. (2020) 35:2801–3. 10.1007/s11606-020-05991-z 32583345 PMC7314426

[23] Guan WJ Liang WH Zhao Y Liang HR Chen ZS Li YM. Comorbidity its impact on 1590 patients with COVID−19 in China: a nationwide analysis. Eur Respir J. (2020) 55:5. 10.1183/13993003.01227-2020 32217650 PMC7098485

[24] Kim DH Park HC Cho A Kim J Yun KS Kim J. Age–adjusted charlson comorbidity index score is the best predictor for severe clinical outcome in the hospitalized patients with COVID−19 infection. Medicine. (2021) 100:18. 10.1097/MD.0000000000025900 33951004 PMC8104192

[25] Mallapaty S. The coronavirus is most deadly if you are older and male – new data reveal the risks. Nature. (2020) 585:16–7. 10.1038/d41586-020-02483-2 32860026

[26] Gao M Piernas C Astbury NM Hippisley–Cox J O'Rahilly S Aveyard P. Associations between body–mass index and COVID−19 severity in 6.9 million people in England: a prospective, community–based, cohort study. Lancet Diabetes Endo. (2021) 9:350–9. 10.1016/S2213-8587(21)00089-9 33932335 PMC8081400

[27] PHAC. Public Health Agency of Canada. Canadian Chronic Disease Surveillance System (CCDSS), Data Tool 2000–2016, (2018). Canada: PHAo (2019).

[28] de Almeida–Pititto B Dualib PM Zajdenverg L Dantas JR de Souza FD Rodacki M. Severity and mortality of COVID 19 in patients with diabetes, hypertension and cardiovascular disease: a meta–analysis. Diabetol Metab Syndr. (2020) 12:1. 10.1186/s13098-020-00586-4 32874207 PMC7456786

[29] Oliveira MC de Araujo Eleuterio T de Andrade Correa AB da Silva LDR Rodrigues RC de Oliveira BA. Correction to: factors associated with death in confirmed cases of COVID−19 in the state of rio de Janeiro. Bmc Infect Dis. (2021) 21:728. 10.1186/s12879-021-06410-2 34340676 PMC8327052

