# Supplementary material for: Predicting the Risk Factors Associated With Severe Outcomes Among COVID-19 Patients–Decision Tree Modeling Approach
Source: Front Public Health. 2022 May 19;10:838514. doi: 10.3389/fpubh.2022.838514 (PMC9160794; doi:10.3389/fpubh.2022.838514)

**Supplementary Material**

**CTREE models stratified by Age groups (ordinal outcome)**

Supplemental Table 1. CTREE models stratified by Age groups using ordinal outcome (levels=0,1,2,3)

Supplemental Table 2. Performance estimates for CTREE

Supplemental Figure 1. CTREE models stratified by Age groups

1. CTREE model for classifying outcomes among COVID-19 cases: Age groups (18Y-40Y)
2. CTREE model for classifying outcomes among COVID-19 cases: Age groups (41-60Y)
3. CTREE model for classifying outcomes among COVID-19 cases: Age groups (>60Y)

**Performance metrics**

Supplemental Figure 2. CTREE model (bivariate outcome)

a) CTREE model (bivariate outcome) for all age groups

b) CTREE model (bivariate outcome) for age groups (18Y-40Y)

c) CTREE model (bivariate outcome) for age groups (41-60Y)

d) CTREE model (bivariate outcome) for age groups (>60Y)

Supplemental Figure 3. ROC curve for the CTREE model for bivariate outcome (test dataset)

1. ROC for the CTREE model (bivariate) for All Ages
2. ROC for the CTREE model (bivariate) for age groups (18Y-40Y)
3. ROC for the CTREE model (bivariate) for age groups (41-60Y)
4. ROC for the CTREE model (bivariate) for age groups (>60Y)

**Supplemental Table 1. CTREE models stratified by Age groups using ordinal outcome (levels=0,1,2,3)**

| **Models stratified by Age group** | | | |
| --- | --- | --- | --- |
|  | **Age groups** | | |
|  | **18-40Y** | **41-60Y** | **> 60Y** |
| Training dataset (n) | 50610 | 32956 | 14731 |
| Test dataset (n) | 21561 | 14036 | 6288 |
| Total sample size | 72171 | 46992 | 21019 |
| Accuracy | 98% | 94% | 76% |

| Bivariate outcome  (0=no hospitalization, ICU or death.  1=any event of hospitalization, ICU or death) | AUC |
| --- | --- |
| All ages | 0.83 [0.82-0.84] |
| 18-40Y | 0.69 [0.67-0.70] |
| 41-60Y | 0.76 [0.74-0.78] |
| >60Y | 0.80 [0.79-0.81] |

**Supplemental Table 2. Performance estimates for CTREE**

**Supplemental Figure 1.**

**a) CTREE model for classifying outcomes among COVID-19 cases: Age groups (18Y-40Y)**


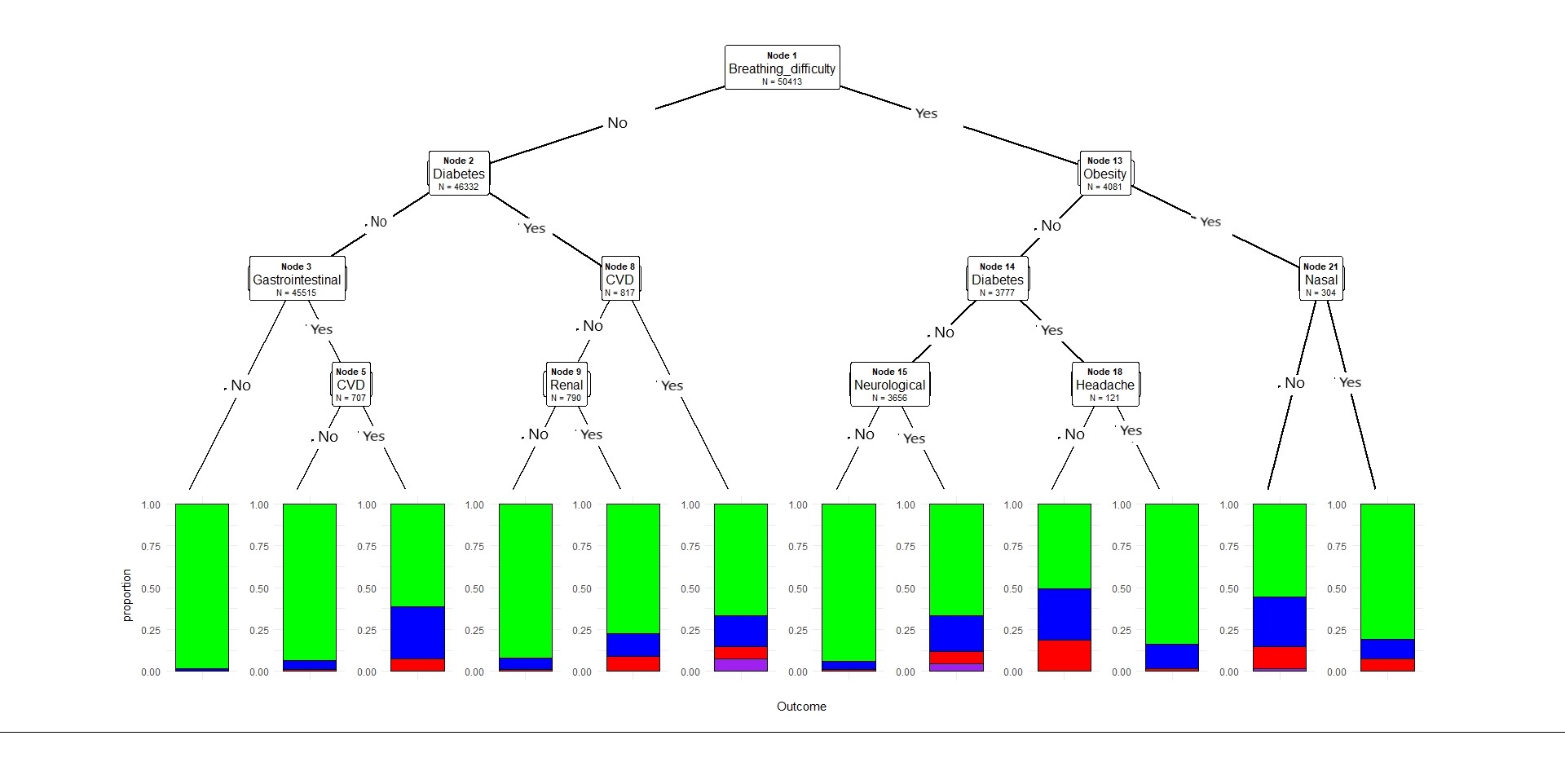


No event

Hospitalization

ICU

Death

**b) CTREE model for classifying outcomes among COVID-19 cases : Age groups (41-60Y)**


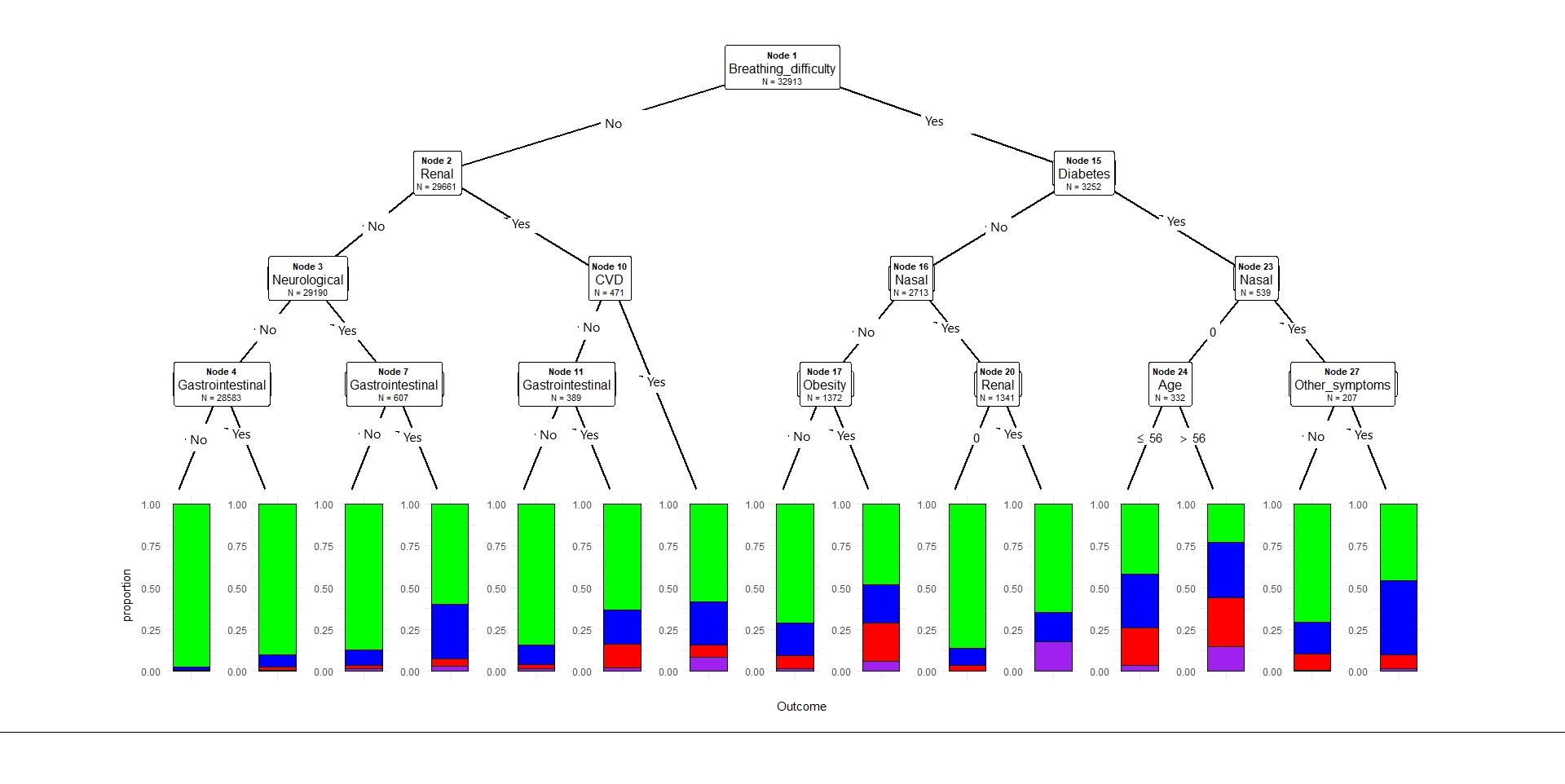


No event

Hospitalization

ICU

Death

**c) CTREE model for classifying outcomes among COVID-19 cases: Age groups (>60Y)**

No event

Hospitalization

ICU

Death

No event

Hospitalization

Death


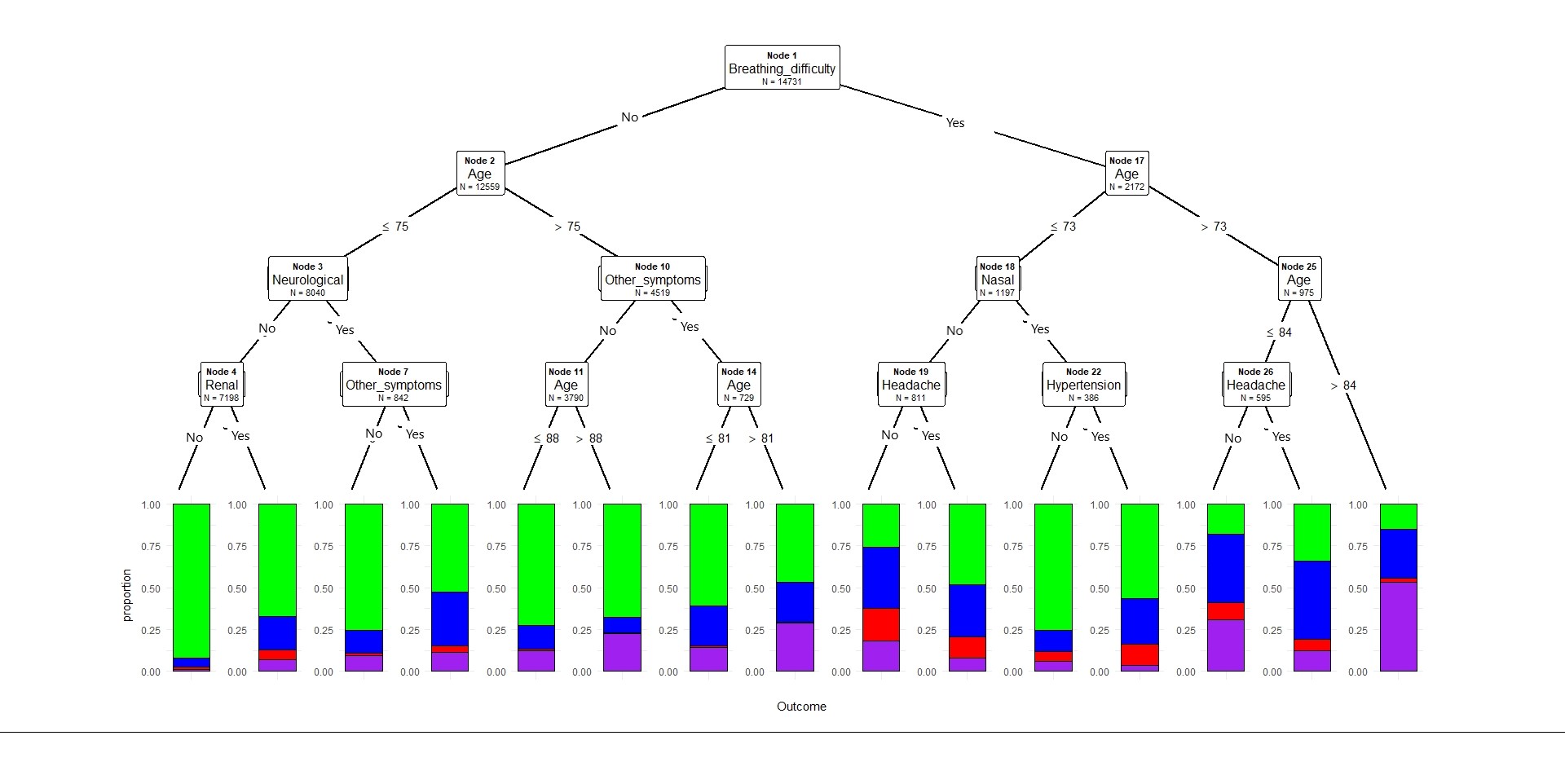


Figure Legend (a-c):

Conditional inference decision tree for classifying severe outcomes (hospitalization, ICU admission death) among COVID-19 positive cases. The outcomes are described in color codes: green color- no hospitalization, ICU or death; blue- hospitalization only, no ICU or death; red- admitted to ICU, but no death; purple -death. The proportion of the events were plotted on the Y-axis at each node.

**Performance metrics**

The most commonly used approach to estimate the model performance is using the ROC analysis and estimates based on confusion matrix.

In original model the outcome variable is ordinal and has 4 levels (0=no hospitalization, ICU or death; 1= hospitalization only but no ICU or death; 2= admitted to ICU, but no death; 3 =death irrespective of hepatization or ICU). Generating ROC curves for multiclass outcome variable is complex and not easy to interpret. To overcome this technical challenge, we created a binary outcome for the same dataset (0=no hospitalization, ICU or death; 1=any event of hospitalization, ICU or death) and same predictor variables from the original model is used.

We modelled the binary outcome and the predictor variable as described below.

Binary_Outcome ~ age+Sex+Zone+Smoking_status+CVD+Renal+Gastrointestinal+Pulmonary+Neurological+Diabetes+Cancer+Other+Hypertension+Obesity+Fever+Breathing_difficulty+Chest_pain+Headache+Cough+Sore_throat+Pain+Gastrointestinal_symptoms+Nasal+Taste_smell+Other_symptoms

The CTREE model is presented in Supplemental figure 2a-d. The top predictors variables in this model are similar compared to original models with multiclass outcome. ROC analysis is presented in the Supplemental figure 3 and AUC values for the models are available in Table 2.

**Supplemental Figure 2.**


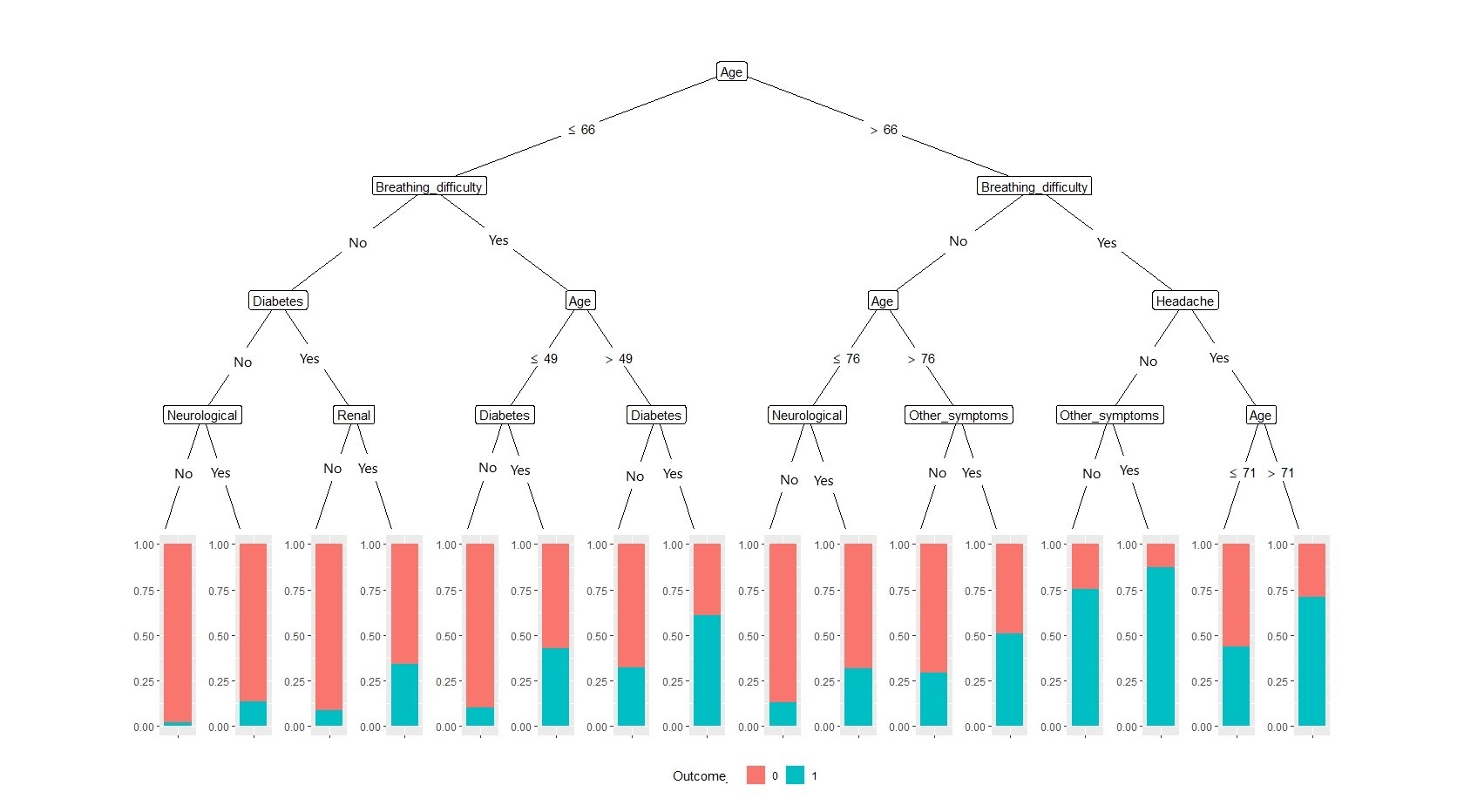
**a) CTREE model (bivariate outcome) for all age groups**


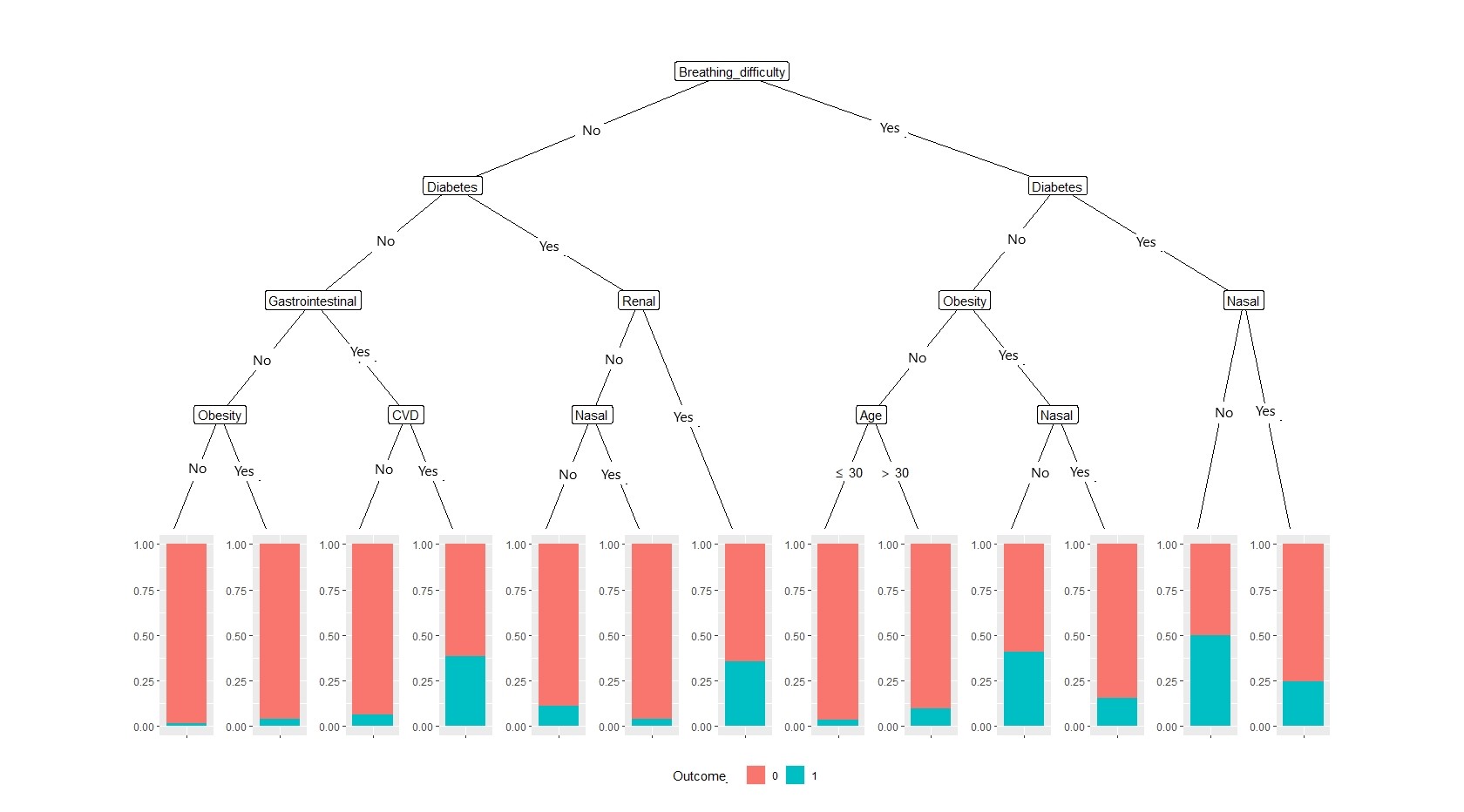
**b) CTREE model (bivariate outcome) for age groups 18-40Y**

c) **CTREE model (bivariate outcome) for age groups 41-60Y**


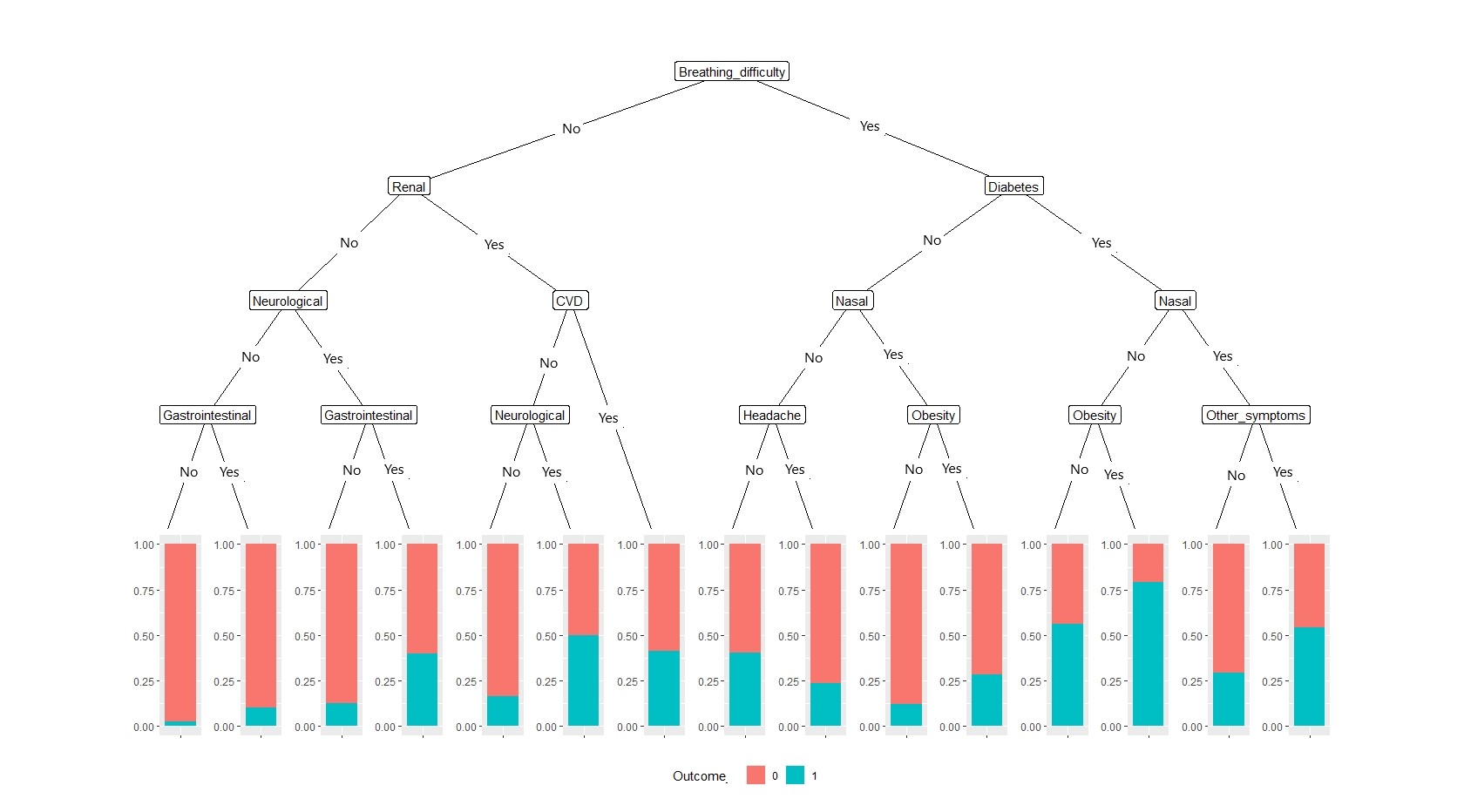


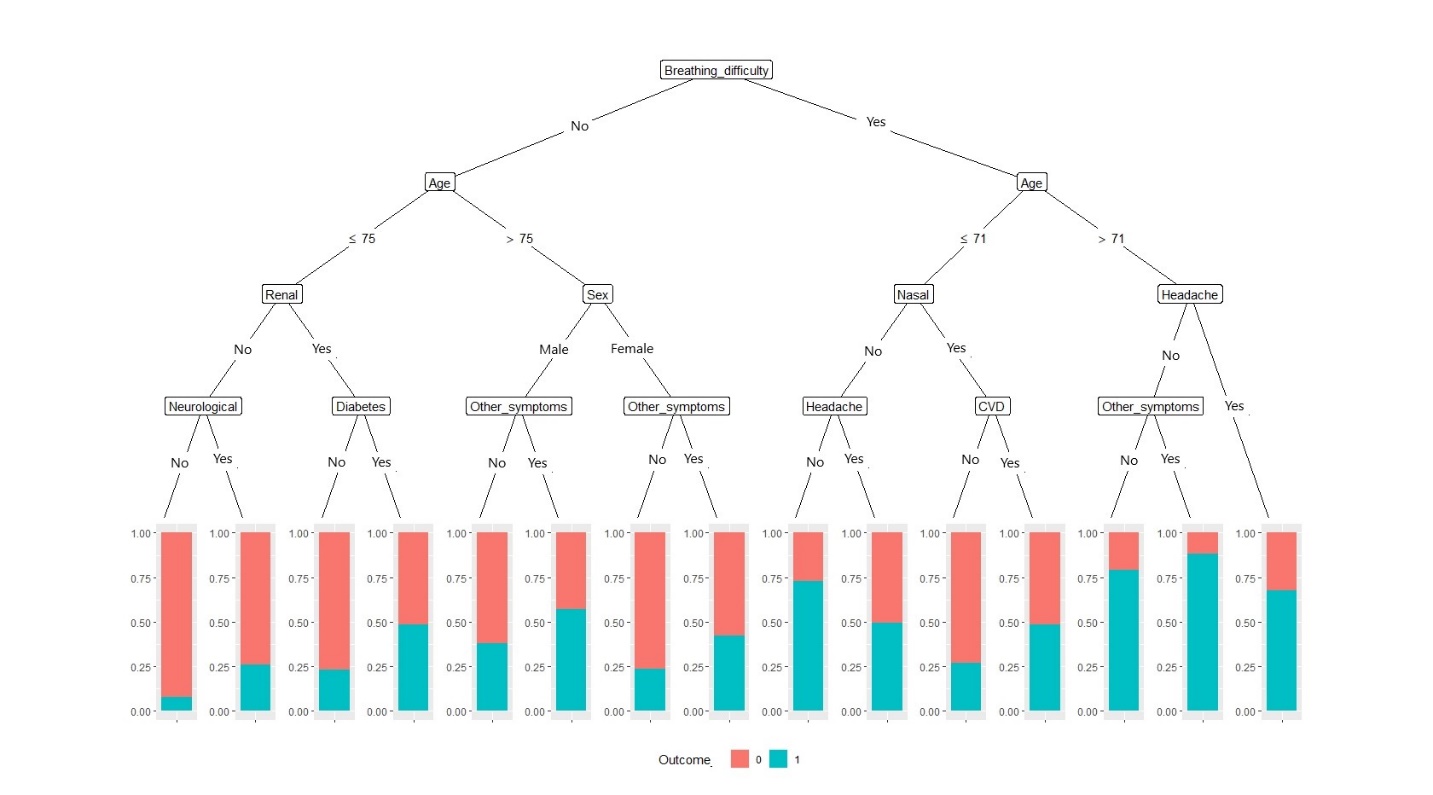
d) **CTREE model (bivariate outcome) for age groups >60 Y**

**Supplemental Figure 3.**

**ROC curve for the CTREE model for bivariate outcome (test dataset)**

1. ROC for the CTREE model (bivariate) for All Ages
2. ROC for the CTREE model (bivariate) for ages 18-40Y


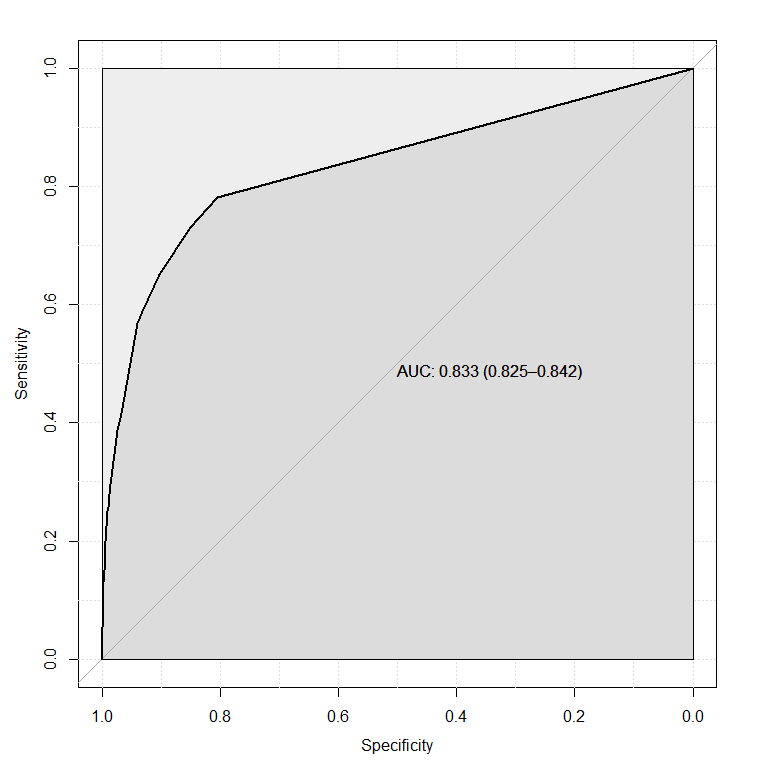

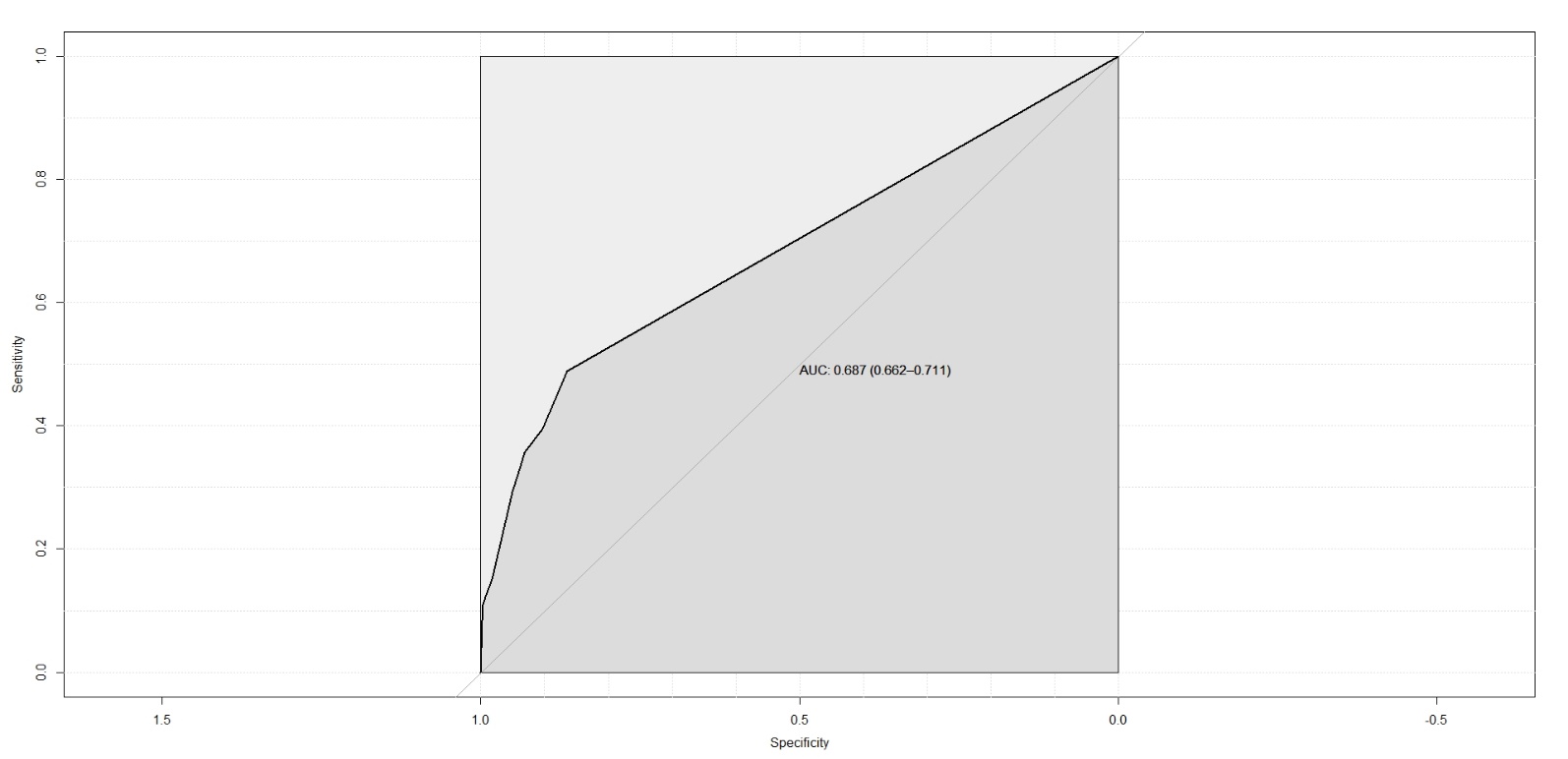


1. ROC for the CTREE model (bivariate) for age 41-60Y
2. ROC for the CTREE model (bivariate) for age >60Y


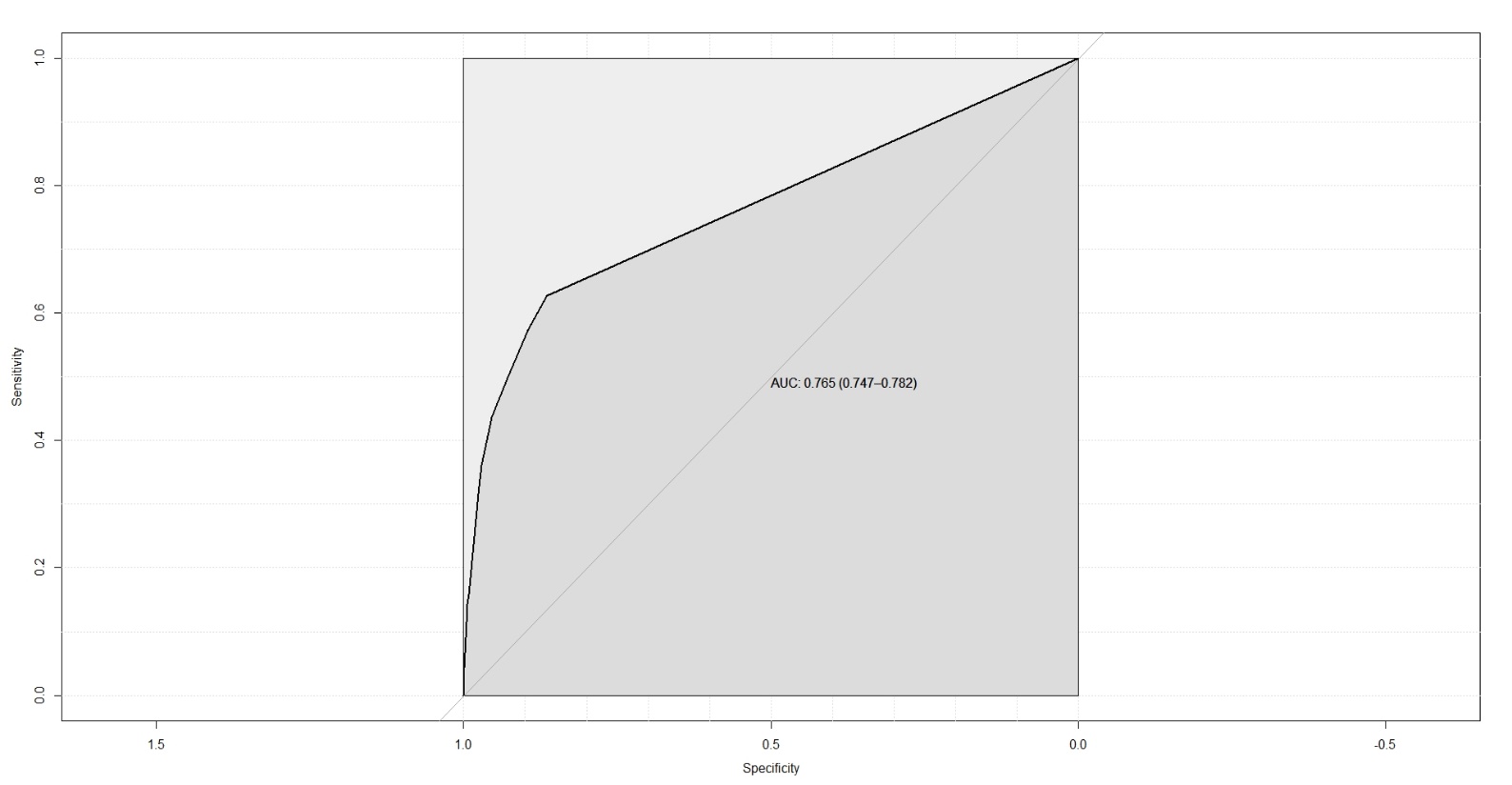

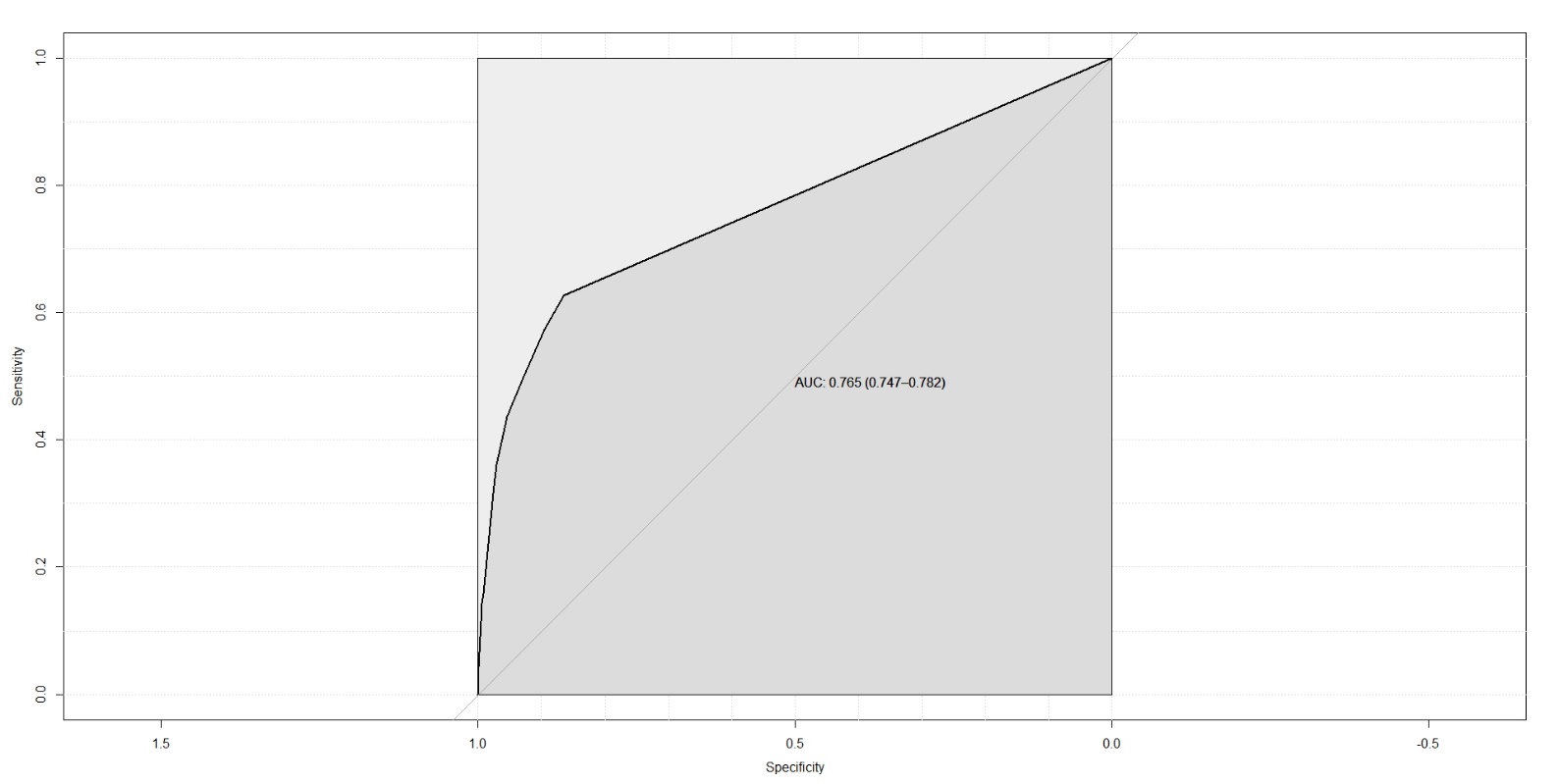

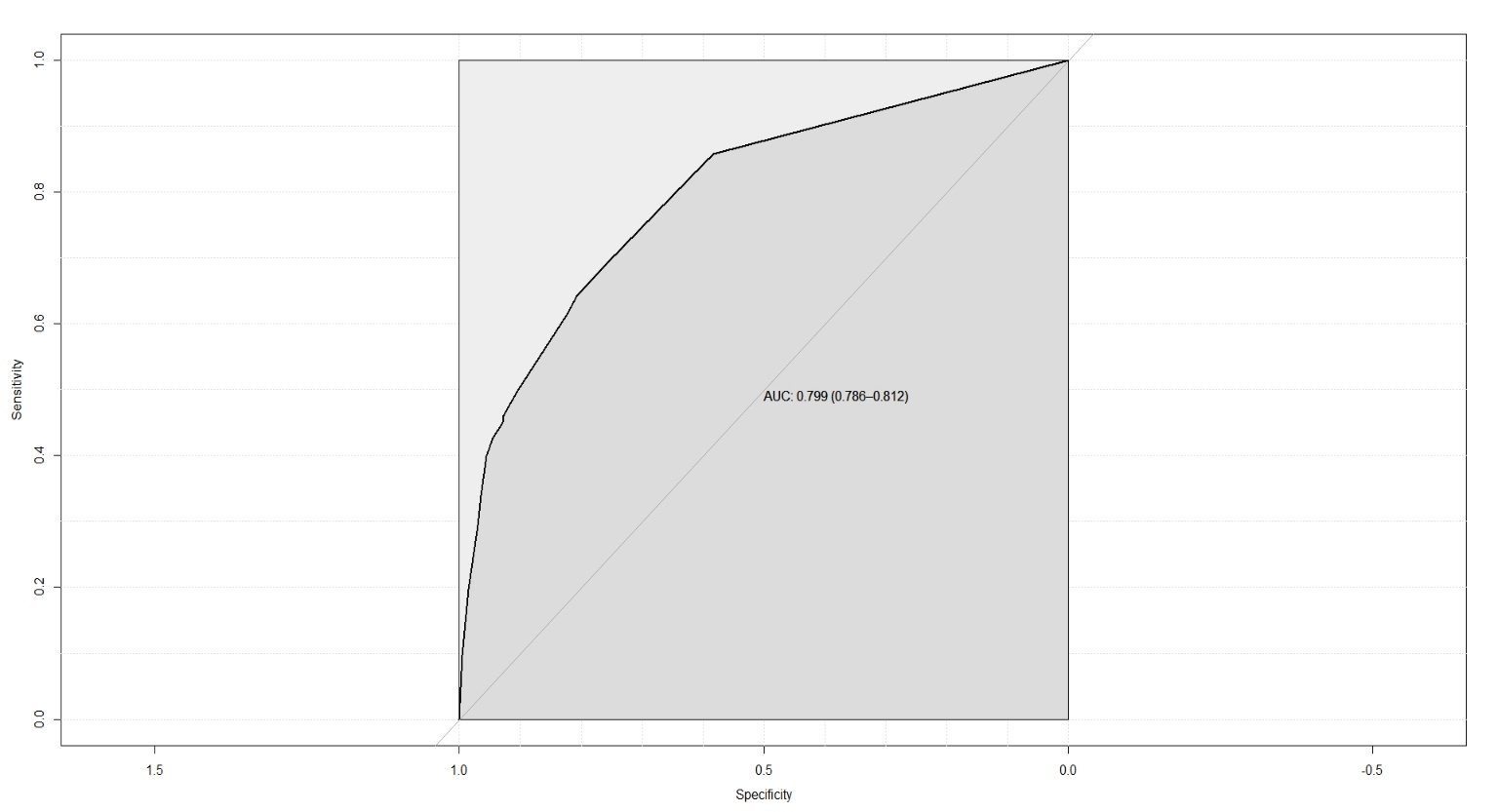

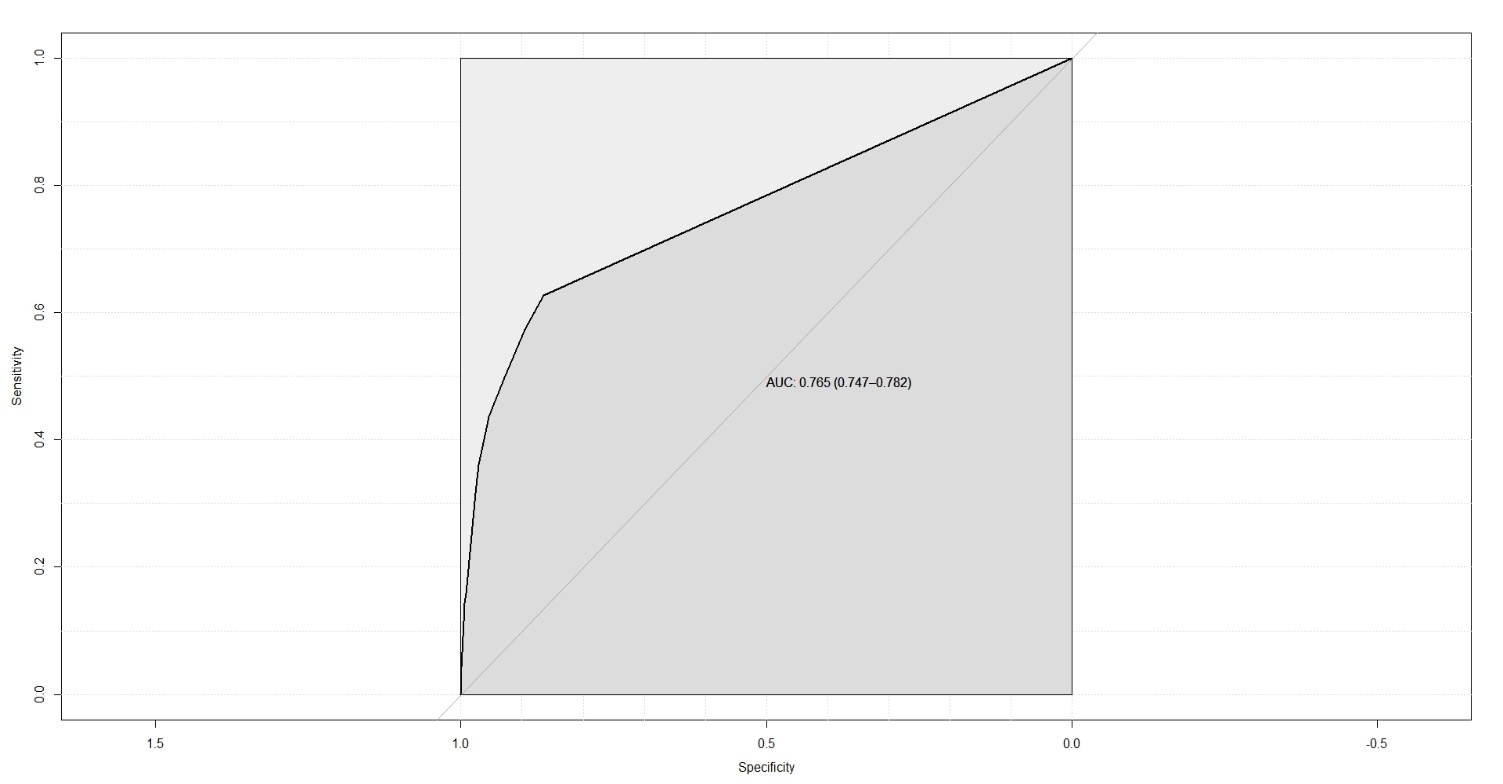

Supplement: Supplementary file 1 [file Data_Sheet_1.DOCX]
